# Effectiveness and economic analysis of the whole cell/recombinant B subunit (WC/rbs) inactivated oral cholera vaccine in the prevention of traveller's diarrhoea

**DOI:** 10.1186/1471-2334-9-65

**Published:** 2009-05-16

**Authors:** Rosa López-Gigosos, Pedro Garcia-Fortea, Maria J Calvo, Emilia Reina, Rosa Diez-Diaz, Elena Plaza

**Affiliations:** 1 International Vaccination Centre of Malaga, Ministry of Health, Subdelegation in Malaga, Paseo Marítimo Pablo Ruiz. Picasso 43, 29017 Málaga (Spain); 2 Dept. Preventive Medicine and Health Public. Malaga University, Malaga, Spain; 3 International Vaccination Centre of Santander Ministry of Health, Delegation in Cantabria, Cantabria, Spain

## Abstract

**Background:**

Nowadays there is a debate about the indication of the oral whole-cell/recombinant B-subunit cholera vaccine (WC/rBS) in traveller's diarrhoea. However, a cost-benefit analysis based on real data has not been published.

**Methods:**

A cost-effectiveness and cost-benefit study of the oral cholera vaccine (WC/rBS), Dukoral^® ^for the prevention of traveller's diarrhoea (TD) was performed in subjects travelling to cholera risk areas. The effectiveness of WC/rBS vaccine in the prevention of TD was analyzed in 362 travellers attending two International Vaccination Centres in Spain between May and September 2005.

**Results:**

The overall vaccine efficacy against TD was 42,6%. Direct healthcare-related costs as well as indirect costs (lost vacation days) subsequent to the disease were considered. Preventive vaccination against TD resulted in a mean saving of 79.26 € per traveller.

**Conclusion:**

According to the cost-benefit analysis performed, the recommendation for WC/rBS vaccination in subjects travelling to zones at risk of TD is beneficial for the traveller, regardless of trip duration and visited continent.

## Background

Travellers' diarrhoea (TD) is rather defined by circumstances of acquisition than by specific microbial agents. TD is usually defined as the passage of 3 or more unformed stools in a 24-hour period, or any number of loose stools if accompanied by abdominal pain, fever, nausea or vomiting. TD is the most frequent syndrome among travellers in most of the visited regions and affects 20–60% of travellers [1]. Some authors have described that 8% of travellers seek medical care upon their return; of these, one third reports diarrhoeal diseases [2-5] TD typically occurs during the first week after arrival, is often self-limiting, and lasts three to four days. Only approximately 2–3% of TD persists longer than a month [1,6]

Efforts to determine the etiology of travellers' diarrhoea in returning travellers encounter several difficulties. Most cases of travellers' diarrhoea are relatively mild and self-limiting, and the patient may not visit a doctor to report it. However, if the patient is ill enough to see a doctor, stool samples are often not obtained for laboratory confirmation. And finally, if a sample is taken and analyzed, it may be impossible to identify a responsible organism. In fact, it has been estimated that only 1 in 136 cases of gastrointestinal infections in the UK is reported to routine surveillance systems [7]. The most common cause of TD worldwide is enterotoxigenic *Escherichia Coli *(ETEC), which induce watery diarrhoea associated with cramps and with low grade or absent fever [1]. ETEC infections are common when there is a breakdown in sanitation, which is often the case in developing countries [5]. Other bacterial etiologies are *Campylobacter *(*jejuni, coli*), *Salmonella*, *Shigella*, *Vibrio cholerae*, *V. parahaemolyicus*, *V. vulnificus*, *Yersinia enterocolitica*, and *Clostridium difficile *[1,8]. Because most cases of cholera are mild or moderate [1,9-11]., one part of TD contracted in cholera-endemic or epidemic countries may be cholera [12].

The most important determinant of risk is the travel destination. Regional differences in both the risk and etiology of diarrhoea divide the world into three grades of risk (high, intermediate, and low). High-risk areas include most of Asia, the Middle East, Africa, and Central and South America. Approximately 50,000 daily cases of TD are estimated among the 50 million people travelling to developing countries. More temperate regions involve seasonal variations in diarrhoea risk. In South Asia, for example, much higher TD attack rates are commonly reported during the hot pre-monsoon months [4]

TD occurs equally in males and females, and is more common in young adults than in older people. Others risk factors for TD include anti-acid medications, achlorhydria, hypoclorhydria, gastrectomy, type O blood, or immune deficiency [6] In short-term travellers, bouts of TD do not appear to protect against future attacks, and more than one episode of TD may occur during a single trip. For travellers to high-risk areas, several approaches may be recommended which can minimize, but never completely eliminate, the risk of TD. The usual recommendations about basic hygiene are usually quickly forgotten, and followed only by a small number of travellers. Therefore, besides these useful recommendations, complementary actions for controlling these diseases -mainly transmitted by contaminated water and food- should be considered [13]

In Spain, preventive care of international travellers (health education and vaccination) is mostly performed through a network of 52 public International Vaccination Centres (IVC). This network was visited by a total of 188,445 and 204,985 travellers during 2005 and 2006, respectively [14]. IVC belong to a state-based network, which performs travellers' care only. The consultations peak is from the month of May to September. By late June 2005, the oral vaccine Dukoral^® ^(whole-cell/recombinant B-subunit cholera vaccine, WC/rBS) was marketed in Spain subsequent to its authorization in the European Union for the prevention of cholera, in April 2004 [15]. Dukoral^® ^had previously been authorized in another 25 countries for the indication of both cholera and ETEC-related diarrhoea, except in Australia, where it was authorized for cholera prevention only [16]. Several studies have evidenced the efficacy of WC/rBS vaccine for protection against diarrhoea caused by LT-related ETEC [17-19].

The objectives of this article were:

1. To assess the effectiveness of WC/rBS cholera vaccine in the prevention of diarrhoea in subjects travelling to cholera endemic-epidemic zones and having attended the Spanish network of IVC.

2. To perform economical (cost-effectiveness and cost-benefit) analysis of WC/rBS vaccination in subjects travelling to high-risk zones of TD, as compared to non-vaccinated subjects with a similar destination.

## Methods

### Vaccine effectiveness

This study was designed as a retrospective cohort study, performed by means of a phone survey to 362 subjects having travelled to zones at risk of cholera and TD.

The WC/rBS cholera vaccine is indicated in subjects travelling for more than 7 days to cholera-endemic or epidemic countries, or else in shorter stays if the trip or the traveller involves high-risk circumstances. Traveller's diarrhoea was considered to be any diarrhoeal process, as defined in the introduction and perceived as such by the traveller, occurring during a journey or within 7 days after the return.

Non-vaccinated travellers were those attending the IVC before the vaccine was marketed and available, or declining recommendation for vaccine, or failing to attend the centre early enough for proper vaccination.

One cohort of cholera vaccinated subjects (N = 171) and one cohort of non-vaccinated subjects (N = 191) travelling to the same zones were studied. All subjects travelling to cholera risk zones were selected consecutively, namely, 125 travellers (58 vaccinated and 67 non-vaccinated) attending Santander's IVC between July and September 2005, and 237 travellers (113 vaccinated and 124 non-vaccinated) attending Málaga's IVC between May and September 2005. Neither the IVC attending staff nor the traveller during his/her trip were aware of the possibility of being part of a study. After obtaining consent of the travellers, they were interviewed by telephone regarding the trip for which they attended the IVC: travel characteristics, vaccine administration, possible TD occurrence, and corresponding treatment. The interviewer was different in each IVC, being a worker of the same centre visited by the traveller. The phone interview lasted 10 minutes on average per traveller, and all contacted travellers accepted and were interested in taking part in the study (except for one traveller of Santander's IVC). The designed questionnaire contained 40 variables, focusing on personal data (age, sex, medical history), trip details and, in vaccinated travellers, confirming that they had taken Dukoral^® ^correctly and whether they experienced any side effect. The questionnaire also asked about TD occurrence during the trip or within the week thereafter, its duration and severity, whether any treatment or medical assistance was required, and TD-related limitation of activity. The mean time interval between the trip and the interview was 54 days (52% were interviewed between two and four weeks after return, 30% between five and eight, and 18% between nine and twenty two weeks), and there were no statistically significant differences between vaccinated and non-vaccinated travellers groups.

The possible bias derived from variability between the two observers was assessed by means of a concordance study. The kappa's index obtained for the TD presence/absence variable was 0.859 (95% CI: 0.591–1.126), corresponding to a 'perfect' or 'substantial' agreement according to the six-category system proposed by Landis and Koch [20].

### Economic analysis (cost-effectiveness and cost-benefit)

#### Model description

The cost and the benefits of the cohort vaccinated against cholera (N = 171) were compared with those of the non-vaccinated cohort (N = 191). Economic analysis was carried out from the healthcare provider's perspective (cost-effectiveness analysis) and from the social perspective (cost-benefit analysis). When analysis was carried out from the provider's perspective, only direct costs and benefits were included. When analysis was done from the social perspective, direct and indirect costs and benefits were included.

The time horizon of the programme was established in 7 months. Such a short time horizon makes unnecessary to apply a discount rate to the costs and benefits. All costs and benefits were expressed in 2005 euros.

To analyze disease evolution with and without the vaccination programme, a decision tree was designed to include all possible events. When the net present value was > 0 the vaccination was considered as money-saving and therefore the cost-effectiveness ratio (being < 0) was not calculated. If the net present value was < 0, a cost-effectiveness analysis was performed.

### Health care cost

All travellers included in the study received healthcare at the IVCs. The cost of healthcare provided at the IVCs was estimated from the figure published by the WHO as cost per visit to health centre by Spanish populations residing within 1 hour of the centre (21.57 € of the year 2000) [21]. This figure was updated to 2005 by means of annual CPI (24.90 €). In travellers not vaccinated with WC/rBS, only healthcare cost (24.90 €) was applied.

### Vaccination cost

The travellers of the vaccinated cohort received two oral doses of the WC/rBS vaccine with a minimum interdose interval of one week. The cost of the two doses of vaccine was 32.31 €.

### Cost of disease in vaccinated and non-vaccinated subjects

#### Direct costs

- Cost of TD treatment: As established by WHO [22], and depending on TD severity, mild cases only require Oral Rehydration Salts (ORS). If unresolved, antibiotics (quinolones or Azithromycin) and antiperistaltics (Loperamide) are added. Treatment with Loperamide (10 capsules, 3.21 €), Azithromycin (3 tablets, 9.68 €) or the recent alternative of Rifaximin (12 tablets, 9.61 €), and Oral Rehydration Salts (5 sachets, 2.33 €, to prepare 5 liters of serum for 2 days of treatment) was assessed as follows:

- Mild TD: ORS (€ × days of TD duration)

- Moderate TD: Loperamide (3.21 €) + Azithromycin or Rifaximin (9.61 €) + ORS (€ × days of TD duration)

The hypothesized cost difference between vaccinated and non-vaccinated subjects lies in the difference of TD duration observed in previous studies [23].

#### Indirect costs

The cost for TD-related lost vacation days is derived from the average cost of 1 vacation day in a organized trip: 142.86 €. This figure results from dividing 3000 € by 21 days (mean cost of a 3-week trip, as usually performed by the study travellers).

### Statistical analysis

The data were processed and analyzed using the Statistical Package for the Social Sciences (SPSS) v.17 (SPSS Inc., Chicago, IL, USA).

### Ethical considerations

Our study was supervised by the Ethics committee of the University of Malaga.

## Results

### Effectiveness of WC/rBS cholera vaccine (Dukoral) in the prevention of TD

All interviewed travellers from vaccinated cohorts took the vaccine properly, and none reported drug-related side effects.

The main findings observed in each Centre's individual analyses are consistent with those obtained by summing the data of both IVC, which are presented below.

1. The frequency of TD in the vaccinated and non-vaccinated travellers groups was 21.1% and 36.6%, respectively, being the difference statistically significant (p = 0.001). This corresponds to a risk difference (RD) of 0.16 (95% CI: 0.07–0.25) and a number needed to treat (NNT) of 6.25 (95% CI: 4–14.3). The relative risk (RR), as a measure of protection against TD, was 0.57 (95% CI: 0.41–0.81). Overall vaccine efficacy was 42.6% (95% CI: 18.9–59.3), with no differences found between the two IVCs (42.9% and 42.5% in Málaga and Santander, respectively).

2. Among vaccinated subjects, TD lasted 1 or 2 days in 72.2% of them and 3 or more days in the remaining 27.8%; while TD duration in non-vaccinated subjects was of 2 days at most in 45.7% of them and longer in the remaining 54.3% (p = 0.009). The mean duration of TD was 2.57 days and 3.59 days in vaccinated and non-vaccinated subjects, respectively.

3. The differences in TD frequency were more relevant in trips to Africa, with TD incidence in vaccinated and non-vaccinated subjects of 16% and 32%, respectively (p = 0.009; Table 1).

**Table 1:** TD frequency and vaccine efficacy (VE) according to destination, age, and trip duration, in vaccinated and non-vaccinated subjects.

	Vaccinated		Non-vaccinated			
	N	TD (%)	N	TD %	p value	VE (%)
Destination						
Africa	100	16.0	90	32.2	0.009	50.3
C and S America	27	29.6	38	36.8	0.545	19.6
South East Asia	44	27.3	63	42.9	0.099	36.4
Age (years)						
< 30	54	24.1	50	54.0	0.002	55.4
30–45	77	24.7	99	31.3	0.212	21.2
> 45	40	10.0	42	28.6	0.031	65.0
Trip duration (Weeks)						
< 3	83	16.9	124	33.1	0.010	49.0
≥ 3	88	25.0	67	43.3	0.016	42.2

4. The protective effect of the vaccine against TD was maintained regardless of trip duration (Table 1). However, vaccine efficacy was somewhat higher in trips of less than 21 days. No significant differences in the vaccine efficacy concerning the time lapse between the return and the interview were found.

5. In order to assess the possible confounding effect of these variables on the relationship between vaccination and TD, a multivariate analysis by logistic regression was performed. For that purpose, 2 categories were considered for traveller's age (between 30 and 44 years, vs. 45 or older), trip duration (20 days or less vs. 21 days or longer), IVC (Málaga vs. Santander), and visited region (Africa vs. other regions). The estimated non-adjusted effect yields an OR of 0.45 (95% CI 0.27–0.73), i.e. a globally protective value. The model that best fits the data includes trip duration as a covariate, and shows that the protective effect of vaccination against TD is slightly increased when adjusted by it (OR = 0.42, 95% CI 0.26–0.69).

### Cost-effectiveness analysis of vaccine recommendation to subjects travelling to zones at high risk of TD

The prevention of traveller's diarrhoea is the assessed effect.

Cost analysis was performed from the perspective of the health care system. Programme costs are all those associated to the use of health technology and involving sacrifice of resources for either the health sector or the patient him/herself.

TD treatment costs have been calculated based on the cost ratio detailed in the 'Material and Methods' section. In the whole sample, 93% of travellers with TD deemed it as 'mild', while only 7% considered it was 'moderate'. None of the interviewed travellers with TD deemed it as 'severe'. No differences in this perception of TD intensity were found between the vaccinated and non-vaccinated cohorts. Treatment costs were calculated according to these ratios.

1.-Treatment of mild TD (93% of subjects with TD) with ORS alone: 2.33 € for each vaccinated subject (5 liters of ORS) and 4.66 € for each non-vaccinated subject (10 liters of ORS).

2.-Treatment of moderate TD (7% of subjects with TD) with ORS + Azithromycin or Rifaximin + Loperamide: 15.15 € for each vaccinated subject (2.33+9.61+3.21) and 17.48 € for each non-vaccinated subject (4.66+9.61+3.21).

As per the above, the mean price of TD treatment was 3.22 € in vaccinated subjects (after weighting 2.33 € for 93%, and 15.15 € for the remaining 7%) and 5.55 € in non-vaccinated subjects (after weighting 4.66 € for 93%, and 17.48 € for the remaining 7%).

The costs associated with adverse events were not considered because vaccine trials had shown that the vaccine was safe and adverse events, if any, would have been negligible.

The expected effectiveness in a theoretical cohort of 1000 vaccinated and non-vaccinated travellers, according to the results of the retrospective study, is shown in Table 2. Applied costs and analysis in theoretical cohorts of 1000 vaccinated and non-vaccinated travellers are shown in Table 3 and Additional file 1.

**Table 2:** Expected incidence of health events in the theoretical cohort of 1000 travellers without and with vaccination.

Vaccine efficacy (%)	TD non-vaccinated	TD vaccinated	Health benefits
*Punctual estim*.	*Expected cases*	*Expected cases*	*Absolute reduction of cases*
42.6 (18.9–59.3)	366 (325–385)	211 (186–237)	155 (133–178)

Note: 95% Confidence interval are expressed between parenthesis

**Table 3:** Unit costs (base case) of estimated cost-generating events.

	Type of events	Cost type	Estimated cost per unit (€)
Health care	Care provided by IVC^1^	Indirect	24.90
Vaccination	Vaccine	Direct	32.31
TD	Treatment in non-vaccinated	Direct	5.55^2^
	Treatment in vaccinated	Direct	3.22^2^
	Lost day of vacations	Indirect	142.86
	Non-vaccinated (142.86 × 3.59)	Indirect	512.86^2^
	Vaccinated (142.86 × 2.57)	Indirect	367.14^2^

^1 ^IVC: International Vacunation Centre

^2^Estimated cost by episode

In cost-effectiveness analysis, the mean cost of each vaccinated and non-vaccinated traveller was 57.88 € and 26.93 €, respectively. This difference correlates basically with the price of the vaccine, given that TD treatment is cheap (particularly in its mild -and most frequent- form). Because multivariant analysis (logistic regression models) allowed detecting that the protective effect of vaccination against TD is increased when trip duration is considered, uncertainty adjustment with regard to this variable was performed. In trips of less than 3 weeks, the mean cost per cholera vaccinated and non-vaccinated traveller was 57.28 € and 29.02 €, respectively. In trips of 3 weeks or more, 56.21 € and 28.04 €, respectively. Differences vs. the whole sample were minimal.

Costs and benefits for theoretical cohorts are gathered in Table 4. As shown, the costs of the vaccination programme were higher than the economic benefits of the programme from the provider perspective (net present value of -30,958.12 €). Although vaccination does not save money, cost-effectiveness ratios are very low.

**Table 4:** Costs and benefits from provider perspective and societal perspective

	Perspective	
Costs per 1000 travellers	Provider	Societal
Vaccination (Vaccine+IVC)	57,210.00	57,210.00
Disease without vaccination	26,931.30	214,637.01
Disease with vaccination	57,889.42	135,356.56
Net saving	-30,958.12	79,280.45
Benefit-cost ratio	-	1.39

### Cost-benefit analysis of vaccine recommendation to tourists travelling to zones at high risk of TD

The number of prevented days of TD is the assessed effect.

Cost analysis was performed from the society perspective. Programme costs are all those associated to the use of health technology (likely to involve sacrifice of resources for the patient), as well as the opportunity cost borne by the patient on account of TD. Direct costs derived from vaccine cost and attention at IVC, as well as the cost of TD treatment, are included (Tables 4 and 5, and Additional file 1). Because the travellers of our study are basically 'tourists' -with only 3.3% of patients travelling for business-, expenses derived from TD-related lost workdays were discarded and those derived from lost vacation days were considered (estimated according to that described in the 'Material and Methods' section). The costs associated with adverse events were not considered because vaccine trials had shown that the vaccine was safe and adverse events, if any, would have been negligible. Given the short term considered for assessing effects and costs, no temporal adjustment was performed.

**Table 5:** Sensitivity analysis, with travel region as main parameter affecting the results.

	Provider perspective	Societal perspective	Benefit-Cost	
	Net present value (€)	Net present value (€)	ratio	Δ
Base case	-30,958.12	79,280.45	1.39	
Region of travel				
*Africa*	-31,036.87	75,474.24	1.32	↓ 4.8%
*C-S. America*	-31,219.34	48,944.96	0.86	↓ 38.3%
*India and SE. Asia*	-30,809.61	88,856.44	1.55	↑ 12.1%

Because vaccination benefits would depend not only on vaccine efficacy but also on the risk of TD borne by the travellers, uncertainty adjustment according to visited region was performed (Table 5). The values of TD incidence considered were those found in the study performed to analyze vaccine effectiveness, shown in Table 1.

From the society perspective the net present value was positive (79,260.14 €) and the cost-benefit ratio was 1.39. Because the net present value was > 0, cost-effectiveness was < 0 and therefore cost-effectiveness ratios were not calculated (Table 4).

Preventive vaccination against TD resulted in a mean saving of 79.26 € per traveller. Depending on the destination (Table 5), this figure would range between 48.94 € and 88.86 €, thereby justifying the indication of WC/rBS vaccination in subjects travelling to zones at risk of TD, regardless of visited continent.

## Discussion

Several potential limitations of this study were associated with its design. This was a non randomized retrospective cohort study and its findings may be affected by recall bias. Interviewing techniques and the questionnaire quality were meticulous to minimize recall bias as it has been suggested [24]. Data was collected in the same way and at similar timing for both vaccinated and not vaccinated travellers. These findings must be considered in light of the methodologic limitations of retrospective recall.

Our study confirms the effectiveness of WC/rBS vaccine against TD described by other authors [5,13,19,25-27] Although relatively low, such effectiveness is in turn important given the frequency of this multicausal pathology called TD. The fact that the vaccine efficacy is greater in our study than in studies by other authors could arise from the proportion of travellers to the west coast of Africa, where several outbreaks of cholera took place during 2005, as well as from the higher proportion of young adults who participated in the study, since the risk for diarrhoea is higher among this age cohort.

The efficacy of vaccination against cholera is high (85%) and its impact on public health is very positive [28,29]., given the associated mortality in endemic zones and epidemic periods. The efficacy of WC/rBS against the ensemble of TDs is logically lower because the vaccine prevents TDs caused by *Vibrio cholerae *and by LT- ETEC, even by ETEC combined with *Salmonella enterica *[19], but fails to do so with the high number of remaining pathogens.

Vaccine recommendation is clear in subjects travelling to cholera zones, as well as in those travelling to zones at risk of TD who suffer from previous conditions where TD may have serious consequences [5,30-32].

Convenient, however, is the economic analysis of the general recommendation for preventive vaccination against TD, from the perspectives of both the traveller and the public health systems. Indeed, on account of the unceasing increase in travellers, the cost of health care provided to travellers due to travel-related diseases is increasingly high for the different health systems. Registers and studies measuring the expenses associated to these pathologies are scarce. For instance, the United Kingdom has vigilance systems of traveller diseases, and studies have been published which evidence that the cost of travel-related illnesses in the UK is in excess of € 11 million, and that, by far, the commonest afflictions the traveller is likely to experience are diarrhoea and vomiting [33]. Hard data are lacking, not only on the cost effectiveness and cost benefit of prevention and treatment of TD [31], but also on their health situation after travel (such as how many returning travellers are ill due to TD, what percentage need medical care at home, and how long they are absent from work), unit costs and total healthcare costs broken down to specific disease groups [34]. For these reasons, many assumptions and extrapolations have to be made that can potentially lead to flawed estimates [34]. According to Thomson and Booth, it must be noted that the financial benefit-cost ratio of an intervention may not be the most suitable measure of its desirability. Any possible treatment will have a better benefit-cost ratio than preventive measures. In economic terms, however, vacation days are usually much more valued by travellers than the economic value of the trip's price. The difficulty to quantify the value of the absence of disease is a limiting but obvious factor in any economic analysis.

The analysis performed from the healthcare provider's perspective has a reasonable cost (57.89 € per traveller vaccinated with WC/rBS, vs. 26.96 € per non-vaccinated travellers). This cost is barely relevant in the ensemble of trip expenses and in the traveller's view to avoid a condition which, banal as it may be, is unpleasant. The cost-benefit analysis is clearly positive, and would be even better if analysis were extrapolated to business trips, where 'lost workdays' costs would have to be added.

Also relevant in this study is that all calculations of the economic analysis are applied on the effectiveness data of our observational study. Further prospective studies with bigger samples and including traveller groups of different profiles (visitors to friend and relatives, business people, etc.) may add to the present assessment. A limitation to the present study is the fact that all studied travellers came from only two IVCs out of 52 possible centres. The size of the sample, however, has yielded useful results. The costs handled in the study are likely to vary among countries, thereby only allowing approximate, non-exact extrapolation.

## Conclusion

In our study, the effectiveness of cholera vaccine WC/rBS in the prevention against TD was 42.6% (TD ratio reduced by vaccination). Moreover, the mean duration of TD in vaccinated travellers with the disease is shortened. According to the cost-benefit analysis performed, the recommendation for WC/rBS vaccination in subjects travelling to zones at risk of TD is beneficial for the traveller, regardless of trip duration and visited continent.

## Competing interests

The authors declare that they have no competing interests.

## Authors' contributions

RLG: conceived of the specific study as described in this paper, coordinated data collection, performed the statistical analyses, and drafted the manuscript. PGF and MJC: contributions to design and revising the manuscript for important content. ER, RDD and EP: participated in the collection of the data, and helped to draft the manuscript. All authors participated in revising the final manuscript.

## Pre-publication history

The pre-publication history for this paper can be accessed here:

http://www.biomedcentral.com/1471-2334/9/65/prepub

## Supplementary Material

Additional file 1 **Supplementary table S1**. Costs (€) of vaccination and disease in the theoretical cohort of 1000 travellers without and with vaccination, from the provider and societal perspectives. Click here for file
