# Supplementary material for: Effectiveness and economic analysis of the whole cell/recombinant B subunit (WC/rbs) inactivated oral cholera vaccine in the prevention of traveller's diarrhoea
Source: BMC Infect Dis. 2009 May 16;9:65. doi: 10.1186/1471-2334-9-65 (PMC2686703; doi:10.1186/1471-2334-9-65)
Supplement: Additional file 1 — Supplementary table S1. Costs (€) of vaccination and disease in the theoretical cohort of 1000 travellers without and with vaccination, from the provider and societal perspectives. [file 1471-2334-9-65-S1.doc]

Table 4. Costs (€) of vaccination and disease in the theoretical cohort of 1000 travellers without and with vaccination, from the provider and societal perspectives

|  | Provider perspective | | | | | | | Societal perspective | | | | | | |
| --- | --- | --- | --- | --- | --- | --- | --- | --- | --- | --- | --- | --- | --- | --- |
|  | Non-vaccinated | | | Vaccinated | | | Saving | Non-vaccinated | | | Vaccinated | | | Saving |
|  | N | cost/U | cost total | N | cost/U | cost total |  | N | cost/U | cost total | N | cost/U | cost total |  |
| IVC care | 1000 | 24.90 | 24900.00 | 1000 | 24.90 | 24900.00 | 0 | 1000 | 24.90 | 24900.00 | 1000 | 24.90 | 24900.00 | 0 |
| Vaccine | 0 | 32.31 | 0 | 1000 | 32.31 | 32310.00 | -32310.00 | 0 | 32.31 | 0 | 1000 | 32.31 | 32310.00 | -32310.00 |
| TD costs |  |  |  |  |  |  |  |  |  |  |  |  |  |  |
| TD treatment | 366 | 5.55 | 2031. 3 | 211 | 3.22 | 679.42 | 1351.88 | 366 | 5.55 | 2031. 3 | 211 | 3.22 | 679.42 | 1351.88 |
| Lost vacation | 0 | 512.86 | 0 | 0 | 367.14 | 0 | 0 | 366 | 512.86 | 187705.71 | 211 | 367.14 | 77467.14 | 110238.5 |
| Total costs |  |  | 26931. 3 |  |  | 57889.42 | -30958.12 |  |  | 214616.1 |  |  | 135356.56 | 79280.45 |
